# Supplementary material for: Pyruvate Dehydrogenase Kinase 1 inhibition mediated oxidative phosphorylation enhancement in cartilage promotes osteoarthritis progression
Source: BMC Musculoskelet Disord. 2023 Jul 20;24:597. doi: 10.1186/s12891-023-06585-6 (PMC10357736; doi:10.1186/s12891-023-06585-6)
Supplement: Supplementary file 1 — Additional file 1. [file 12891_2023_6585_MOESM1_ESM.zip › BP.Gsea.1653623667859/GOBP_SMOOTH_MUSCLE_CELL_DIFFERENTIATION.html]

Details for gene set GOBP\_SMOOTH\_MUSCLE\_CELL\_DIFFERENTIATION[GSEA]

|  || Dataset | 55457\_collapsed\_to\_symbols.cls.cls #OA\_versus\_normal.cls.cls #OA\_versus\_normal\_repos |
| Phenotype | cls.cls#OA\_versus\_normal\_repos |
| Upregulated in class | normal |
| GeneSet | GOBP\_SMOOTH\_MUSCLE\_CELL\_DIFFERENTIATION |
| Enrichment Score (ES) | -0.44380015 |
| Normalized Enrichment Score (NES) | -1.7058084 |
| Nominal p-value | 0.0016977929 |
| FDR q-value | 0.12895969 |
| FWER p-Value | 1.0 |
Table: GSEA Results Summary

  

Fig 1: Enrichment plot: GOBP\_SMOOTH\_MUSCLE\_CELL\_DIFFERENTIATION      
 Profile of the Running ES Score & Positions of GeneSet Members on the Rank Ordered List

  

| SYMBOL | TITLE | RANK IN GENE LIST | RANK METRIC SCORE | RUNNING ES | CORE ENRICHMENT || 1 | EFEMP2 | EGF containing fibulin extracellular matrix protein 2 [Source:HGNC Symbol;Acc:HGNC:3219] | 156 | 0.853 | 0.0322 | No |
| 2 | COMP | cartilage oligomeric matrix protein [Source:HGNC Symbol;Acc:HGNC:2227] | 415 | 0.700 | 0.0485 | No |
| 3 | SGCB | sarcoglycan beta [Source:HGNC Symbol;Acc:HGNC:10806] | 687 | 0.597 | 0.0584 | No |
| 4 | GPER1 | G protein-coupled estrogen receptor 1 [Source:HGNC Symbol;Acc:HGNC:4485] | 1677 | 0.402 | 0.0023 | No |
| 5 | FGF9 | fibroblast growth factor 9 [Source:HGNC Symbol;Acc:HGNC:3687] | 1833 | 0.380 | 0.0100 | No |
| 6 | SIX1 | SIX homeobox 1 [Source:HGNC Symbol;Acc:HGNC:10887] | 2011 | 0.360 | 0.0149 | No |
| 7 | SOX9 | SRY-box transcription factor 9 [Source:HGNC Symbol;Acc:HGNC:11204] | 2089 | 0.351 | 0.0272 | No |
| 8 | KIT | "KIT proto-oncogene, receptor tyrosine kinase [Source:HGNC Symbol;Acc:HGNC:6342]" | 2117 | 0.348 | 0.0431 | No |
| 9 | DNMT1 | DNA methyltransferase 1 [Source:HGNC Symbol;Acc:HGNC:2976] | 2836 | 0.267 | 0.0011 | No |
| 10 | NFATC1 | nuclear factor of activated T cells 1 [Source:HGNC Symbol;Acc:HGNC:7775] | 3027 | 0.247 | -0.0008 | No |
| 11 | NFATC3 | nuclear factor of activated T cells 3 [Source:HGNC Symbol;Acc:HGNC:7777] | 3079 | 0.242 | 0.0078 | No |
| 12 | ACVR1 | activin A receptor type 1 [Source:HGNC Symbol;Acc:HGNC:171] | 3898 | 0.165 | -0.0473 | No |
| 13 | HEY2 | hes related family bHLH transcription factor with YRPW motif 2 [Source:HGNC Symbol;Acc:HGNC:4881] | 4058 | 0.150 | -0.0519 | No |
| 14 | MEF2C | myocyte enhancer factor 2C [Source:HGNC Symbol;Acc:HGNC:6996] | 4218 | 0.135 | -0.0572 | No |
| 15 | EDNRA | endothelin receptor type A [Source:HGNC Symbol;Acc:HGNC:3179] | 4663 | 0.099 | -0.0866 | No |
| 16 | TBX3 | T-box transcription factor 3 [Source:HGNC Symbol;Acc:HGNC:11602] | 4790 | 0.088 | -0.0919 | No |
| 17 | MED28 | mediator complex subunit 28 [Source:HGNC Symbol;Acc:HGNC:24628] | 5187 | 0.057 | -0.1197 | No |
| 18 | TMEM204 | transmembrane protein 204 [Source:HGNC Symbol;Acc:HGNC:14158] | 5229 | 0.054 | -0.1201 | No |
| 19 | GATA6 | GATA binding protein 6 [Source:HGNC Symbol;Acc:HGNC:4174] | 5537 | 0.030 | -0.1424 | No |
| 20 | PDCD4 | programmed cell death 4 [Source:HGNC Symbol;Acc:HGNC:8763] | 5961 | -0.002 | -0.1753 | No |
| 21 | ENG | endoglin [Source:HGNC Symbol;Acc:HGNC:3349] | 7213 | -0.097 | -0.2676 | No |
| 22 | RAMP2 | receptor activity modifying protein 2 [Source:HGNC Symbol;Acc:HGNC:9844] | 7247 | -0.099 | -0.2650 | No |
| 23 | FGFR2 | fibroblast growth factor receptor 2 [Source:HGNC Symbol;Acc:HGNC:3689] | 7374 | -0.108 | -0.2692 | No |
| 24 | ANKRD17 | ankyrin repeat domain 17 [Source:HGNC Symbol;Acc:HGNC:23575] | 7385 | -0.109 | -0.2643 | No |
| 25 | BMP4 | bone morphogenetic protein 4 [Source:HGNC Symbol;Acc:HGNC:1071] | 7816 | -0.142 | -0.2904 | No |
| 26 | APLNR | apelin receptor [Source:HGNC Symbol;Acc:HGNC:339] | 8001 | -0.155 | -0.2966 | No |
| 27 | ZEB1 | zinc finger E-box binding homeobox 1 [Source:HGNC Symbol;Acc:HGNC:11642] | 8147 | -0.166 | -0.2993 | No |
| 28 | HEY1 | hes related family bHLH transcription factor with YRPW motif 1 [Source:HGNC Symbol;Acc:HGNC:4880] | 8213 | -0.172 | -0.2954 | No |
| 29 | MECP2 | methyl-CpG binding protein 2 [Source:HGNC Symbol;Acc:HGNC:6990] | 8687 | -0.208 | -0.3214 | No |
| 30 | SHH | sonic hedgehog signaling molecule [Source:HGNC Symbol;Acc:HGNC:10848] | 8941 | -0.227 | -0.3293 | No |
| 31 | SIRT1 | sirtuin 1 [Source:HGNC Symbol;Acc:HGNC:14929] | 8947 | -0.227 | -0.3178 | No |
| 32 | FOXF1 | forkhead box F1 [Source:HGNC Symbol;Acc:HGNC:3809] | 9933 | -0.315 | -0.3781 | No |
| 33 | EREG | epiregulin [Source:HGNC Symbol;Acc:HGNC:3443] | 10778 | -0.403 | -0.4229 | Yes |
| 34 | ITGA8 | integrin subunit alpha 8 [Source:HGNC Symbol;Acc:HGNC:6144] | 11046 | -0.437 | -0.4209 | Yes |
| 35 | CTH | cystathionine gamma-lyase [Source:HGNC Symbol;Acc:HGNC:2501] | 11108 | -0.444 | -0.4026 | Yes |
| 36 | NFATC4 | nuclear factor of activated T cells 4 [Source:HGNC Symbol;Acc:HGNC:7778] | 11363 | -0.481 | -0.3974 | Yes |
| 37 | ADM | adrenomedullin [Source:HGNC Symbol;Acc:HGNC:259] | 11543 | -0.506 | -0.3850 | Yes |
| 38 | MRTFA | myocardin related transcription factor A [Source:HGNC Symbol;Acc:HGNC:14334] | 11613 | -0.518 | -0.3635 | Yes |
| 39 | MRTFB | myocardin related transcription factor B [Source:HGNC Symbol;Acc:HGNC:29819] | 11668 | -0.527 | -0.3403 | Yes |
| 40 | TBX2 | T-box transcription factor 2 [Source:HGNC Symbol;Acc:HGNC:11597] | 11728 | -0.538 | -0.3169 | Yes |
| 41 | EDNRB | endothelin receptor type B [Source:HGNC Symbol;Acc:HGNC:3180] | 12075 | -0.613 | -0.3120 | Yes |
| 42 | FOXO4 | forkhead box O4 [Source:HGNC Symbol;Acc:HGNC:7139] | 12240 | -0.656 | -0.2907 | Yes |
| 43 | WNT4 | Wnt family member 4 [Source:HGNC Symbol;Acc:HGNC:12783] | 12331 | -0.686 | -0.2621 | Yes |
| 44 | PDGFB | platelet derived growth factor subunit B [Source:HGNC Symbol;Acc:HGNC:8800] | 12389 | -0.704 | -0.2299 | Yes |
| 45 | SRF | serum response factor [Source:HGNC Symbol;Acc:HGNC:11291] | 12549 | -0.773 | -0.2021 | Yes |
| 46 | NOTCH1 | notch receptor 1 [Source:HGNC Symbol;Acc:HGNC:7881] | 12555 | -0.776 | -0.1622 | Yes |
| 47 | VEGFA | vascular endothelial growth factor A [Source:HGNC Symbol;Acc:HGNC:12680] | 12629 | -0.813 | -0.1257 | Yes |
| 48 | HES1 | hes family bHLH transcription factor 1 [Source:HGNC Symbol;Acc:HGNC:5192] | 12676 | -0.849 | -0.0852 | Yes |
| 49 | SOD2 | superoxide dismutase 2 [Source:HGNC Symbol;Acc:HGNC:11180] | 12685 | -0.853 | -0.0415 | Yes |
| 50 | CTNNB1 | catenin beta 1 [Source:HGNC Symbol;Acc:HGNC:2514] | 12866 | -1.119 | 0.0026 | Yes |
Table: GSEA details [plain text format]

  

Fig 2: GOBP\_SMOOTH\_MUSCLE\_CELL\_DIFFERENTIATION      
 Blue-Pink O' Gram in the Space of the Analyzed GeneSet

  

Fig 3: GOBP\_SMOOTH\_MUSCLE\_CELL\_DIFFERENTIATION: Random ES distribution      
 Gene set null distribution of ES for **GOBP\_SMOOTH\_MUSCLE\_CELL\_DIFFERENTIATION**
